# Thermodynamic and Kinetic Stabilities of Al(III) Complexes with N_2_O_3_ Pentadentate Ligands

**DOI:** 10.3390/molecules28093764

**Published:** 2023-04-27

**Authors:** Edoardo Callegari, Jonathan Martinelli, Nicol Guidolin, Mariangela Boccalon, Zsolt Baranyai, Lorenzo Tei

**Affiliations:** 1 Department of Science and Technological Innovation, Università del Piemonte Orientale, Viale T. Michel 11, 15121 Alessandria, Italy; edoardo.callegari1996@gmail.com (E.C.); jonathan.martinelli@uniupo.it (J.M.); 2 Bracco Research Centre, Bracco Imaging S.p.A., Via Ribes 5, 10010 Colleretto Giacosa, Italy; nicol.guidolin@bracco.com (N.G.); mariangela.boccalon@bracco.com (M.B.)

**Keywords:** aluminum, fluoride, polydentate chelators, thermodynamics, kinetic inertness

## Abstract

Al(III) complexes have been recently investigated for their potential use in imaging with positron emission tomography (PET) by formation of ternary complexes with the radioisotope fluorine-18 (^18^F). Although the derivatives of 1,4,7-triazacyclononane-1,4,7-triacetic acid (NOTA) are the most applied chelators for [Al^18^F]^2+^ labelling and (pre)clinical PET imaging, non-macrocyclic, semi-rigid pentadentate chelators having two N- and three O-donor atoms such as RESCA1 and AMPDA-HB have been proposed with the aim to allow room temperature labelling of temperature-sensitive biomolecules. The paucity of stability data on Al(III) complexes used for PET imaging instigated a complete thermodynamic and kinetic solution study on Al(III) complexes with aminomethylpiperidine (AMP) derivatives AMPTA and AMPDA-HB and the comparison with a RESCA1-like chelator CD3A-Bn (*trans*-1,2-diaminocyclohexane-*N*-benzyl-*N*,*N′*,*N′*-triacetic acid). The stability constant of [Al(AMPDA-HB)] is about four orders of magnitude higher than that of [Al(AMPTA)] and [Al(CD3A-Bn)], highlighting the greater affinity of phenolates with respect to acetate O-donors. On the other hand, the kinetic inertness of the complexes, determined by following the Cu^2+^-mediated transmetallation reactions in the 7.5–10.5 pH range, resulted in a spontaneous and hydroxide-assisted dissociation slightly faster for [Al(AMPTA)] than for the other two complexes (*t*_1/2_ = 4.5 h for [Al(AMPTA)], 12.4 h for [Al(AMPDA-HB)], and 24.1 h for [Al(CD3A-Bn)] at pH 7.4 and 25 °C). Finally, the [AlF]^2+^ ternary complexes were prepared and their stability in reconstituted human serum was determined by ^19^F NMR experiments.

## 1. Introduction

Positron emission tomography (PET) is a diagnostic imaging technique that employs chemical tracers containing positron-emitting radionuclides. Fluorine-18 (^18^F) is the most commonly used positron emitter, mainly due to its suitable radiochemical properties and accessibility. The favorable properties of ^18^F include (1) a half-life time of 110 min; (2) its almost pure positron emission (97% β^+^, 3% EC); (3) its decay product, ^18^O, stable isotope; (4) the low energy of the emitted positrons (0.63 MeV) and the short positron range in tissues (1–2 mm in water), allowing the capture of images with good resolution [1,2].

In most cases, fluorinated tracers contain ^18^F bound to a small organic molecule, such as for [^18^F]fluorodeoxyglucose (FDG), which stands out among the ^18^F radiotracers for its superior performance [3]. However, the synthesis of fluorinated tracers requires numerous and often laborious synthetic steps, organic solvents, catalysts, and high temperatures. At the same time, ^18^F is obtained as an aqueous solution by proton irradiation of [^18^O]OH_2_, which considerably reduces the nucleophilicity of the F^−^ ions. Long drying steps, anhydrous aprotic solvents, and high temperatures are required to increase nucleophilicity. Therefore, in the field of PET tracers, there is always a need to find new methods for the rapid and efficient introduction of ^18^F in complex and temperature-sensitive biomolecules.

The coordination approach for the labelling of biomolecules with ^18^F consists in the formation of a strong bond between the fluorine atom and elements such as boron, silicon, or aluminum, requiring a lower activation energy for their formation than that necessary for the formation of C-F bonds. Moreover, Si-F (540 kJ mol^−1^), B-F (766 kJ mol^−1^), and Al-F (664 kJ mol^−1^) [4] are high-energy bonds potentially highly stable for in vivo imaging applications. Furthermore, the main strength of the coordination approach lies in the possibility of carrying out a single radiolabelling reaction, ideally very fast, which can be easily automated for routine production. In addition, being able to operate in an aqueous environment, long and laborious protocols are not required for the anhydrification of the ^18^F ion, further accelerating the preparation process of the radiotracer.

**Figure 1:**
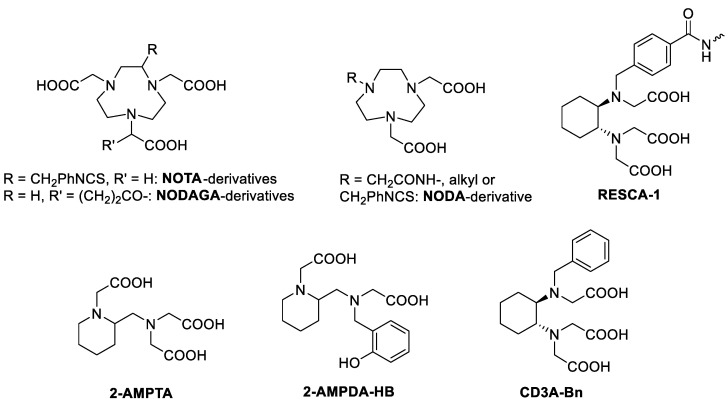
Chelating ligands discussed in the text.

Very recent reviews [5,6,7,8] retrace in detail all the research carried out on aluminum fluoride-based radiotracers since the first publication in 2009 by McBride et al. [9], who were the first to explore the Al^18^F method for the radiolabelling of peptides of pharmaceutical interest. They tested several derivatives of the hexa- and pentadentate macrocyclic ligands based on 1,4,7-triazacyclononane (NOTA and NODA, respectively; see Figure 1) having N_3_O_3_ and N_3_O_2_ coordinative sets of donor atoms for the coordination of [Al^18^F]^2+^, respectively [10,11]. Advanced clinical studies using peptides (Alfatide I and II [12], octreotide [13], neurotensin [14]) conjugated to a macrocyclic chelator labelled with [Al^18^F]^2+^ have been published in recent years for the visualization of neuroendocrine tumors, prostate cancer, and metastases in lung cancer, lymph nodes, or bones. Although these macrocyclic chelators show considerable potential, the high temperature required for the complexation reaction (≥100 °C) is still the main limit to the widespread application of this radiolabelling approach, especially for the labelling of temperature-sensitive biomolecules. Thus, Bormans and co-authors initially used EDTA-based pentadentate chelators [15], obtaining good labelling yields but low stability in physiological conditions and in serum of the [Al^18^F]^2+^ complexes. To increase the kinetic inertness, the rigidity of the chelator was therefore increased using CDTA-like systems (CDTA = *trans*-1,2-diaminocyclohexane-*N*,*N*,*N′*,*N′*-tetraacetic acid). A bifunctional chelator, called RESCA1, was then conjugated to a nanobody or to interleukin-2 and labelled with [Al^18^F]^2+^ at room temperature and tested in vivo with reasonable success [16,17,18]. Our group has recently proposed the use of 2-aminomethylpiperidine (AMP)-based pentadentate chelators for [Al^18^F]^2+^ labelling [19]. In particular, the AMPDA-HB chelator bearing two acetate and one phenolate pendant arms and, therefore, a N_2_O_3_ donor set, showed particular high labelling yields with [Al^18^F]^2+^ at room temperature and 5–6.5 pH range. Moreover, the labelled complex highlighted high stability *in vitro* (up to 4 h in PBS, serum, and EDTA solutions) and in vivo, rapid hepatobiliary and renal excretion, and low accumulation in the bones.

Although over the last decade the [^18^F]AlF approach has become part of the recognized procedures in the field of nuclear medicine and it is actually an effective tool in radiopharmaceutical design [5,6,7,8], there is still a scarcity of data in the literature about the thermodynamic and kinetic properties of the Al^3+^ and AlF^2+^ complexes. It should be highlighted that radiochemists and medical researchers usually focus on radiochemical yields and in vivo stability, but a more detailed knowledge of the physico-chemical properties of the Al(III) complexes itself is also very important before applying these systems in vivo. Only the [Al(NOTA)] and [Al(F)(NOTA)]^−^ systems have been investigated and the thermodynamic stability constant of the Al(III) complex and its dissociation rates in acidic and alkaline solutions reported [20]. Importantly, the recently proposed non-macrocyclic pentadentate N_2_O_3_ chelators for AlF^2+^ complexation (Figure 1) have been successfully labelled with [Al^18^F]^2+^ and some of them tested in vivo, but no data on thermodynamic stability and kinetic inertness of the Al(III) complexes have been yet reported. Thus, in this work, we carried out a detailed characterization of the Al^3+^ complexes with AMPTA, AMPDA-HB, and CD3A-Bn, a non-conjugatable analogue of RESCA1 (Figure 1), using pH potentiometry, UV spectrophotometry, and ^1^H and ^27^Al NMR spectroscopy. A ^19^F NMR study on the mixed AlF^2+^ complexes and their stability in serum is also reported.

## 2. Results and Discussion

The ligands 2-AMPTA and 2-AMPDA-HB were synthesized as reported previously by our group [19,21], whereas CD3A-Bn was synthesized as reported in the literature [22]. The Al^19^F complexes were also prepared by first forming AlF^2+^, mixing AlCl_3_ and NaF in water, followed by the complexation with the specific ligand. Al^19^F complexes were purified by semi-preparative HPLC-MS and characterized by ESI-MS and ^27^Al and ^19^F NMR spectroscopy (see ESI).

### 2.1. Equilibrium Properties of the Al(III)^–^AMPTA, Al(III)-AMPDA-HB, and Al(III)-CD3A-Bn Systems

Since the thermodynamic properties of any metal complex proposed for in vivo applications must be characterized by high thermodynamic stability, the equilibrium properties of AMPTA, AMPDA-HB, and CD3A-Bn ligands and their Al(III) complexes were investigated in detail. First, the protonation constants of the ligands, defined by Equation (1), were determined by pH-potentiometry and the log*K*_i_^H^ values are listed in Table 1.

(1) $$K_{i}^{H}=\frac{\left[H_{i}L\right]}{\left[H_{i-1}L\right]\left[H^{+}\right]}$$

The protonation sequence of AMPTA and AMPDA-HB was investigated by ^1^H NMR spectroscopy and spectrophotometry, respectively [21]. According to the ^1^H NMR studies, first and second protonations of AMPTA took place at the N-atoms of the piperidine moiety and of the iminodiacetic acid group. At pH < 4, further protonation of AMPTA occurs on the carboxylate groups of the iminodiacetic acid [21]. On the other hand, spectrophotometric studies revealed that the first and second protonations of the AMPDA-HB take place at the nitrogen of the aminomethyl group (the protonation occurs partially at the N-atom and the phenolate-O^¯^ group due to the H-bond formation) and the phenolate-O^−^ group. The log*K*_2_^H^ value of AMPDA-HB is comparable with that of phenol (log*K*^H^ = 10.0, 0.1 M NaClO_4_) [23]. Further protonation of AMPDA-HB takes place at the non-protonated piperidine-N and the carboxylate-O donor atoms in the pendant arms [21]. Comparing the log*K*_i_^H^ values of the AMPTA, AMPDA-HB, and CD3A-Bn reveals that the log*K*_1_^H^ value of AMPDA-HB is higher by 0.5 and 1.3 log*K* units than those of AMPTA and CD3A-Bn, which can be explained by the hydrogen bonding between the protonated N and the basic phenolate-O^−^ donor atoms. Moreover, the relatively low log*K*_1_^H^ value of CD3A-Bn is attributed to the small electron withdrawing effect of the benzyl substituent. On the other hand, the log*K*_1_^H^ value of AMPTA, AMPDA-HB, and CD3A-Bn ligands is about 1.2–2.5 log*K* units higher than those of the CDTA and EDTA ligands due to the formation of [Na(CDTA)]^3−^ and [Na(EDTA)]^3−^ complexes ([Na(CDTA)]^3−^: log*K*_NaL_ = 4.66; [Na(EDTA)]^3−^: log*K*_NaL_ = 1.43, 0.5 M Me_4_NCl, 25 °C) [24,25]. The log*K*_i_^H^ values of AMPTA and AMPDA-HB ligands obtained in 0.15 M NaCl and 0.15 M NaNO_3_ solutions are comparable, which indicates that the presence of NO_3_^−^ instead of Cl^−^ has practically no effect for the protonation constants of these pentadentate ligands.

**Table 1:** Protonation constants of ligands, stability, and protonation constants of the Al^III^-complexes formed with AMPTA, AMPDA-HB, CD3A-Bn, and CDTA ligands (25 °C).

	AMPTA		AMPDA-HB		CD3A-Bn	EDTA ^c^	CDTA ^c^
I	0.15 M NaNO_3_	0.15 M NaCl ^a^	0.15 M NaNO_3_	0.15 M NaCl ^a^	0.15 M NaNO_3_	0.15 M NaNO_3_	
log*K*_1_^H^	11.49 (1)	11.67	12.0 (1) ^b^	12.4	10.72 (2)	9.40	9.54
log*K*_2_^H^	5.56 (4)	5.47	9.92 (5) ^b^	10.14	5.22 (3)	6.10	6.08
log*K*_3_^H^	2.75 (5)	2.74	4.94 (5)	4.76	3.34 (3)	2.72	3.65
log*K*_4_^H^	1.73 (5)	1.62	2.01 (5)	1.91	0.59 (9)	2.08	2.69
log*K*_5_^H^	-	-	-	-	-	1.23	1.14
Σlog*K*_1–4_^H^	21.53	21.50	28.87	29.21	19.87	20.30	21.96
Al^III^-complexes							
log*K*_AlL_	14.9 (1) ^d^		18.6 (1) ^d^		14.5 (1) ^d^	16.5 ^e^	18.9 ^e^
log*K*_AlLH_−1__	5.06 (6)		6.94 (6)		5.20 (4)	6.0 ^e^	7.70 ^e^
log*β*_AlLH_−1__	9.8 (1)		11.7 (1)		9.3 (1)	10.5 ^e^	11.2 ^e^

^a^ Ref. [21]; ^b^ Spectrophotometry (0.15 M NaNO_3_, 25 °C); ^c^ Ref. [26]; ^d 27^Al NMR spectroscopy (0.15 M NaNO_3_, 25 °C); ^e^ Ref. [27], 0.1 M KNO_3_, 25 °C.

The stability and protonation constants of the Al(III) complexes of AMPTA, AMPDA-HB, and CD3A-Bn, defined by Equations (2)–(4), were investigated by pH-potentiometry and by multinuclear NMR spectroscopy at 25 °C in 0.15 M NaNO_3_ solution.

(2) $$K_{\mathrm{AlL}}=\frac{\left[\mathrm{AlL}\right]}{\left[{\mathrm{Al}}^{3+}\right]\left[L\right]}$$

(3) $$K_{{\mathrm{AlLH}}_{-1}}=\frac{\left[\mathrm{AlL}\right]}{\left[{\mathrm{AlLH}}_{-1}\right]\left[H^{+}\right]}$$

(4) $${\mathrm{Al}}^{3+}+L+{\mathrm{OH}}^{-}\;⇌{\mathrm{AlLH}}_{-1}$$

$$\beta_{{\mathrm{AlLH}}_{-1}}=\frac{\left[{\mathrm{AlLH}}_{-1}\right]}{\left[{\mathrm{Al}}^{3+}]\left[L\right][{\mathrm{OH}}^{-}\right]}$$

In order to avoid the hydrolysis of the Al(III) ion, the pH-potentiometric titration of the pre-prepared complexes of AMPTA, AMPDA-HB, and CD3A-Bn ligands was performed at pH > 4.0. An extra equivalent base consumption in the pH-potentiometric titration profiles revealed the formation of [Al(AMPTA)H_−1_]^−^, [Al(AMPDA-HB)H_−1_]^−^, and [Al(CD3A-Bn)H_−1_]^−^ species in the pH range 4–8. Since the Al(III) ion with coordination number 6 is complexed by the pentadentate AMPTA, AMPDA-HB, and CD3A-Bn ligands, this process can be interpreted by the coordination of the OH^−^ ion to the Al(III) ion in [Al(AMPTA)], [Al(AMPDA-HB)], and [Al(CD3A-Bn)] complexes according to Equations (3) and (4). In calculating the equilibrium constants, the best fitting of the mL NaOH—pH data was obtained by assuming the formation of AlL and AlLH_−1_ species.

To determine the stability constant of the Al(III) complexes, the ^1^H and ^27^Al NMR spectra of the Al^3+^-AMPTA, Al^3+^-AMPDA-HB, and Al^3+^-CD3A-Bn systems were recorded in the pH range 8.0–12. ^1^H and ^27^Al NMR spectra of the three systems are shown in Figures S1–S6.

**Figure 2:**
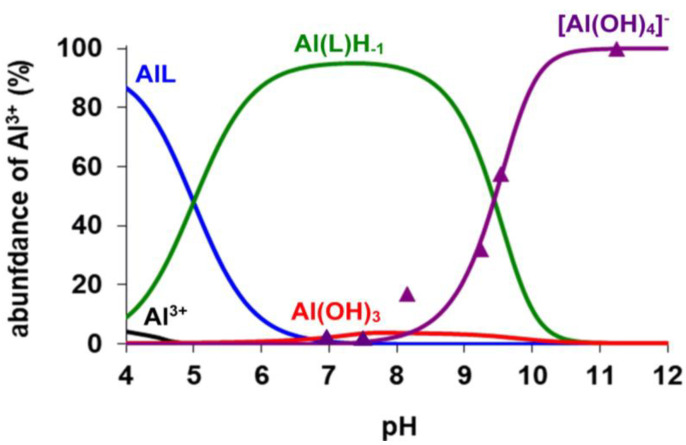
Species distribution and the relative amount of the [Al(OH)_4_]^−^ (▲) determined by ^27^Al NMR spectroscopy in Al^3+^-AMPTA system ([Al^3+^] = [AMPTA] = 5.0 mM, 0.15 M NaNO_3_, 25 °C).

**Figure 3:**
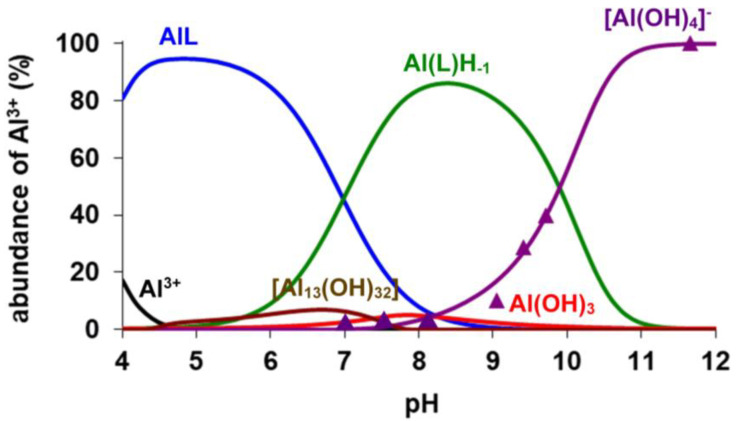
Species distribution and the relative amount of the [Al(OH)_4_]^−^ (▲) determined by ^27^Al NMR spectroscopy in Al^3+^-AMPDA-HB system ([Al^3+^] = [AMPDA-HB] = 5.0 mM, 0.15 M NaNO_3_, 25 °C).

**Figure 4:**
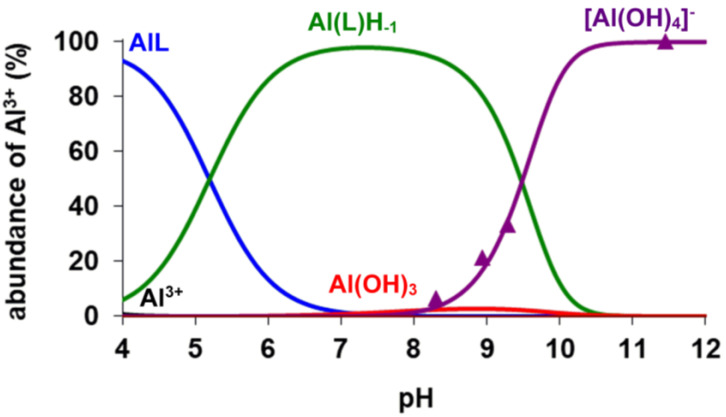
Species distribution and the relative amount of the [Al(OH)_4_]^−^ (▲) determined by ^27^Al NMR spectroscopy in Al^3+^-CD3A-Bn system ([Al^3+^] = [CD3A-Bn] = 4.0 mM, 0.15 M NaNO_3_, 25 °C).

The analysis of the ^27^Al NMR data was used for the calculation of the stability constants of the [Al(AMPTA)], [Al(AMPDA-HB)], and [Al(CD3A-Bn)] complexes. Integrals of the ^27^Al NMR signal ([Al(OH)_4_]^−^ at 81 ppm, ν_½_ = 11 Hz; see Figures S2, S4 and S6) were used for the calculation of the log*K*_AlL_ value of the three Al complexes by taking into account the protonation constant of the AlLH_−1_ species (Equation (3), Table 1) and the known hydrolysis constants of the free Al^3+^ ion (log*K*_[Al(OH)]_^2+^ = −4.60, log*K*_[Al(OH)_2_]_^+^ = −9.09, log*K*_[Al(OH)_3_]_ = −14.94 and log*K*_[Al(OH)_4_]_^−^ = −23.08, log*K*_[Al_2_(OH)_2_]_^4+^ = −8.0, log*K*_[Al_3_(OH)_4_]_^5+^ = −13.47, log*K*_[Al_13_(OH)_32_]_^7+^ = −104.81) [28,29]. As shown in Table 1, the log*K*_AlL_ value of [Al(AMPDA-HB)] is higher by about 3.5–4 log*K* units than that of [Al(AMPTA)] and [Al(CD3A-Bn)]. Interestingly, the stability constant of [Al(AMPDA-HB)] is comparable and even higher by 2.5 log*K* units than the Al(III) complex formed with the hexadentate CDTA and EDTA ligands, explained by the higher basicity of the phenolate-O^−^ and by the higher total basicity of AMPDA-HB (Σlog*K*_1–4_^H^, Table 1). In the Al(III) complexes of the present work, the Al(III) ion is presumably coordinated by 2 amino-N, two or three carboxylate-O^−^, and the very basic phenolate-O^−^ donor atoms, whereas the sixth coordination site of the Al(III) ion is occupied by the inner-sphere water molecule to complete the octahedral coordination geometry [30]. Interestingly, the log*K*_AlLH_−1__ value characterizing the formation of [Al(AMPDA-HB)H_−1_]^−^ is about 1–1.5 log*K* units higher than that of [Al(AMPTA)H_−1_]^−^, [Al(CD3A-Bn)H_−1_]^−^, and [Al(EDTA)H_−1_]^2−^ and somewhat lower than that of [Al(CDTA)H_−1_]^2−^. Presumably, the formation of the [AlLH_−1_] species takes place through the deprotonation of the inner-sphere water molecule directly coordinated to the Al(III) ion. In [Al(AMPDA-HB)], the coordination of the very basic and bulky phenolate-O^−^ can hinder the entrance of the OH^−^ ion to the inner sphere of the Al(III) ion due to the high electron density on the phenolate-O^−^. Moreover, the comparison of the log*K*_AlLH_−1__ values characterizing the formation of [AlLH_−1_] species (Table 1) shows that[Al(AMPDA-HB)H_−1_]^−^ has the highest cumulative stability constant among the Al(III) complexes formed with AMPTA, AMPDA-HB, CD3A-Bn, EDTA, and CDTA ligands. The equilibrium data obtained by pH-potentiometry and multinuclear NMR spectroscopy were used to calculate the species distribution diagram for the Al(III)-AMPTA, Al(III)AMPDA-HB, and Al(III)-CD3A-Bn systems (Figure 1, Figure 2 and Figure 3). The amount of [Al(OH)_4_]^−^ species determined by ^27^Al NMR studies (Figures S2, S4 and S6) is also shown in Figure 2, Figure 3 and Figure 4.

The species distribution diagrams and the ^1^H and ^27^Al NMR spectra (Figure 2, Figure 3 and Figure 4 and Figures S1–S6) indicate that formation of the three Al(III) complexes is completed at pH > 4.5. In the three systems, the [AlLH_−1_] species predominates at pH > 7.0. The ^1^H NMR spectra of all [AlLH_−1_] species contain several sharp multiplets (Figures S1, S3 and S5), which allow us to assume that the Al(III) complexes have a rigid structure due to the tightly coordinated Al(III) ion by the N_2_O_3_ set of donor atoms. The chemical shifts and the linewidth of the ^27^Al NMR signals are strongly influenced by the nature of the donor atoms and by the symmetry of the complexes ([Al(NOTA)]: δ_Al_ = 50 ppm, ν_½_~50 Hz [20]; [Al(EDTA)]^−^ δ_Al_ = 41.2 ppm, ν_½_~975 Hz and [Al(CDTA)]^−^ δ_Al_ = 40.5 ppm, ν_½_~740 Hz) [31]. On the other hand, the linewidth of the ^27^Al NMR signal might be influenced by the interaction of the nuclear quadrupolar moment with the electric field gradient at the ^27^Al nucleus [32]. At pH > 8.0, the competition of AMPTA, AMPDA-HB, and CD3A-Bn with the OH^−^ ion for Al^3+^ is confirmed by the appearance of the ^27^Al NMR signal of [Al(OH)_4_]^−^ (δ_Al_ = 81 ppm, ν_½_ = 11 Hz; see Figures S2, S4 and S6) and of the ^1^H NMR signals of the free AMPTA, AMPDA-HB, and CD3A-Bn ligands (Figures S1, S3 and S5). The intensity of the ^27^Al NMR signal of [Al(OH)_4_]^−^ increases with increasing pH due to the dissociation of [AlLH_−1_] species in the pH range 8.0–11.0. Interestingly, the ^1^H NMR signal of the free AMPTA, AMPDA-HB, and CD3A-Bn ligands has a significant upfield shift due to the deprotonation of the free ligands at pH > 9.0.

### 2.2. Kinetic Inertness of the [Al(AMPTA)], [Al(AMPDA-HB)], and [Al(CD3A-Bn)]

**Figure 5:**
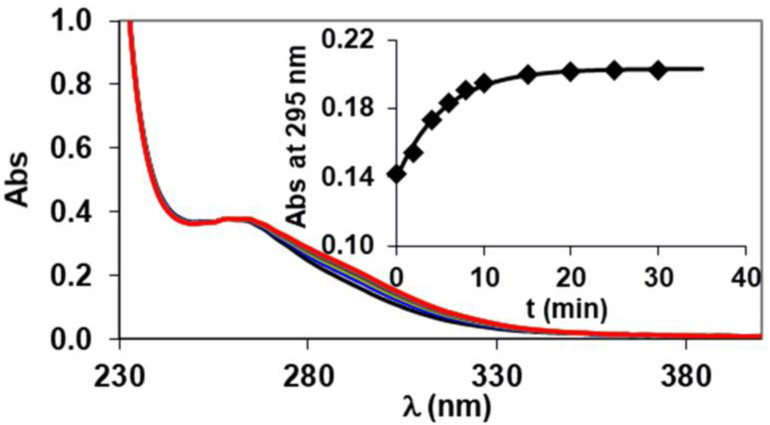
Absorption spectra of the [Al(CD3A-Bn)]-Cu(II)-citrate reacting system at different time points ([AlL] = 0.1 mM, [Cu^2+^] = 0.1 mM, [Cit] = 10 mM, [piperazine] = 10 mM, pH = 9.0, 0.15 M NaCl, 25 °C).

The Al(III) complexes are generally characterized by relatively low thermodynamic stability and high kinetic inertness due to the slow ligand exchange reactions. However, the large excess of the endogenous competition partners (mainly Cu(II) and Zn(II) and/or transferrin) [33] may cause the transmetallation or transchelation reactions of the complexes, which can result in the in vivo release of the Al(III) ion. The kinetic properties of Al(III)-complexes are often measured in strong acidic ([H^+^] > 1.0 M) and basic ([OH^−^] > 0.1 M) conditions [20]. In this study, the kinetic inertness of [Al(AMPTA)], [Al(AMPDA-HB)], and [Al(CD3A-Bn)] was determined by following the Cu^2+^-mediated transmetallation reactions (Equation (5)) with spectrophotometry on the absorption band of the resulting Cu(II) complexes in the presence of 10-fold Cu^2+^ and 100- and 200-fold citrate excess to prevent the hydrolysis of the released Al^3+^ and of the exchanging Cu^2+^ ions in the pH range 7.0–10.5. The absorption spectra of the [Al(CD3A-Bn)]-Cu(II)-citrate reacting system as a representative for the transmetallation reaction between Al(III) complexes and the Cu^2+^ ion in the presence of citrate excess are presented in Figure 5.

(5) AlL+Cu(II) ⇌CuL+Al(III)

**Figure 6:**
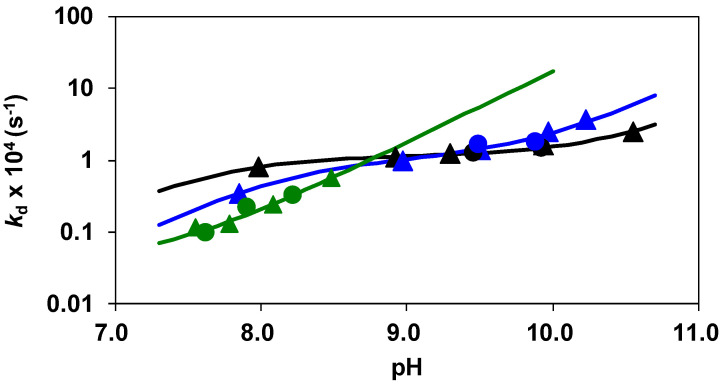
*k*_d_ pseudo-first-order rate constants characterizing the dissociation of the [Al(AMPTA)], [Al(AMPDA-HB)], and [Al(CD3A-Bn)] complexes as a function of pH. The solid lines and the symbols represent the calculated and the experimental *k*_d_ values, respectively. ([Al(AMPTA)]: [AlL] = 0.1 mM, [Cu^2+^] = 1.0 mM, [Cit] = 10 mM (▲) and 20 mM (●); [Al(AMPDA-HB)]: [AlL] = 0.1 mM, [Cu^2+^] = 1.0 mM, [Cit] = 10 mM (▲) and 20 mM (●); [Al(CD3A-Bn)]: [AlL] = 0.1 mM, [Cu^2+^] = 1.0 mM, [Cit] = 10 mM (▲) and 20 mM (●), [HEPES] = [piperazin] = 10 mM, 0.15 M NaCl, 25 °C).

The rates of the metal exchange reactions were studied in the presence of large Cu(II)-citrate excess, so the transmetallation can be treated as a pseudo-first-order kinetic process and the reaction rates can be expressed by Equation (6):

(6) $$-\frac{d{\left[\mathrm{AlL}\right]}_{t}}{\mathrm{dt}}=k_{d}{\left[\mathrm{AlL}\right]}_{t}$$

where *k*_d_ is a pseudo-first-order rate constant and [AlL]_t_ is the total concentration of the Al(III) complexes at the time t, respectively. The pseudo-first-order rate constants characterizing the transmetallation reactions of [Al(AMPTA)], [Al(AMPDA-HB)], and [Al(CD3A-Bn)] with Cu(II) at different pH values in the presence of citrate are shown in Figure 6.

**Scheme 1:**
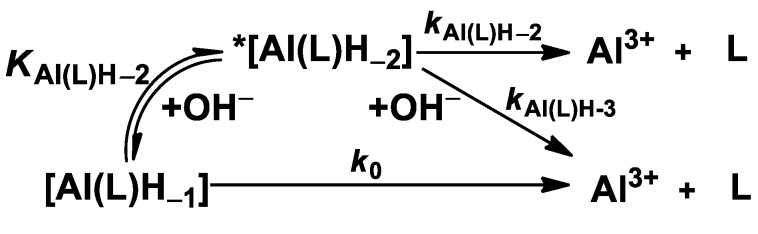
Dissociation mechanism of [Al(AMPTA)], [Al(AMPDA-HB)], and [Al(CD3A-Bn)] complexes.

The *k*_d_ values are independent of the concentration of citrate, which reveals that the concentration of the free citrate and the dominant [Cu(Cit)H_−1_]^2−^ species does not influence the reaction rate. The rate-determining step of the transmetallation reaction is the dissociation of the Al(III) complexes followed by the fast reaction between the free AMPTA, AMPDA-HB, and CD3A-Bn ligands and Cu(II) ions. By taking into account the species distribution of the Al(III) systems (Figure 2, Figure 3 and Figure 4), the Al(L)H_−1_ species predominates in the pH range 7.0–10.5. The *k*_d_ values of [Al(AMPTA)]- Cu(II)-citrate, [Al(AMPDA-HB)]- Cu(II)-citrate, and [Al(CD3A-Bn)]- Cu(II)-citrate reacting systems presented in Figure 6 increase with the pH and show a sigmoid curve for [Al(AMPTA)]- Cu(II)-citrate and [Al(AMPDA-HB)]- Cu(II)-citrate systems and straight line for [Al(CD3A-Bn)]- Cu(II)-citrate system. The dependence of the *k*_d_ values on pH can be interpreted in terms of the spontaneous (*k*_0_, Equation (7)) and OH^−^-assisted (*k*_Al(L)H_−2__, Equation (9) and *k*_Al(L)H_−3__, Equation (10)) dissociation of the Al(III) complex via the formation of the dihydroxy- *[Al(L)H_−2_]^2−^ intermediate (*K*_Al(L)H_−2__, Equation (8)). However, the formation of the *[Al(L)H_−2_]^2−^ intermediate for [Al(CD3A-Bn)] complex cannot be detected, which might be interpreted by the lack or by the lability of the *[Al(CD3A-Bn)H_−2_]^2−^ species. The dissociation of the *[Al(L)H_−2_]^2−^ intermediate formed by [Al(AMPTA)] and [Al(AMPDA-HB)] complexes can also occur by OH^−^ assisted pathway resulting in the increase of the *k*_d_ values in the pH range 9.0–10.5. The mechanisms of the transmetallation reactions for all Al(III) complexes are summarized in Scheme 1.

(7) $$[\mathrm{Al}(L)H_{-1}]\;\overset{k_{0}}{\to }\;{\mathrm{Al}}^{3+}+H_{x}L$$

(8) $$*[\mathrm{Al}(L)H_{-2}]+H^{+}\;⇌[\mathrm{Al}(L)H_{-1}]$$

KAlLH−2=AlLH−1AlLH−2*H+

(9) $$*[\mathrm{Al}(L)H_{-2}]\;\overset{k_{\mathrm{Al}\left(L\right)H_{-2}}}{\to }\;{\mathrm{Al}}^{3+}+H_{x}L$$

(10) $$*[\mathrm{Al}(L)H_{-2}]+{\mathrm{OH}}^{-}\;\overset{k_{\mathrm{Al}\left(L\right)H_{-3}}}{\to }\;{\mathrm{Al}}^{3+}+H_{x}L$$

By taking into account all possible pathways, the rate of the dissociation of the Al(III) complexes can be expressed by Equation (11).

(11) $$-\frac{d\left[\mathrm{AlL}\right]}{\mathrm{dt}}=k_{d}[\mathrm{Al}\left(L\right)H_{-1}{]}_{t}=k_{0}\left[\mathrm{Al}\left(L\right)H_{-1}\right]+k_{\mathrm{Al}\left(L\right)H_{-2}}\left[\mathrm{Al}\left(L\right)H_{-2}\right]+k_{\mathrm{Al}\left(L\right)H_{-3}}\left[\mathrm{Al}\left(L\right)H_{-2}\right][{\mathrm{OH}}^{-}]$$

Considering the total concentration of the complex [AlL]_t_ = [Al(L)H_−1_] + *[Al(L)H_−2_] and the equilibrium constants for the formation of *[Al(L)H_−2_] (*K*_Al(L)H_−2__, Equation (8)), the *k*_d_ pseudo-first-order rate constants presented in Figure 6 can be expressed by Equation (12):

(12) $$k_{d}=\frac{k_{0}+k_{1}\left[{\mathrm{OH}}^{-}\right]+k_{2}{\left[{\mathrm{OH}}^{-}\right]}^{2}}{1+K_{\mathrm{Al}\left(L\right)H_{-2}}\left[{\mathrm{OH}}^{-}\right]}$$

where *k*_0_, *k*_1_ = *k*_Al(L)H_−2__ × *K*_Al(L)H_−2__, and *k*_2_ = *k*_Al(L)H_−3__ × *K*_Al(L)H_−2__ are the rate constants characterizing the spontaneous and OH-assisted dissociation of Al(III) complexes, whereas *K*_Al(L)H_−2__ is the equilibrium constant for the formation of the dihydroxy- *[Al(L)H_−2_]^2−^ intermediate. The rate and equilibrium constants characterizing the transmetallation reaction of [Al(AMPTA)], [Al(AMPDA-HB)], and [Al(CD3A-Bn)] with Cu(II) in the presence of citrate were calculated by fitting the *k*_d_ values presented in Figure 6 to Equation (12).

The rate and protonation constants characterizing the transmetallation reaction of [Al(AMPTA)], [Al(AMPDA-HB)], and [Al(CD3A-Bn)] with Cu^2+^ in the presence of citrate excess are listed in Table 2 and compared with those of [Al(NOTA)].

In the fitting procedure of the kinetic data obtained for the [Al(AMPTA)]-Cu(II)-citrate and [Al(AMPDA-HB)]-Cu(II)-citrate reacting system, the first term of the numerator in Equation (12) was neglected due to the relatively fast OH^−^ assisted dissociation (*k*_Al(L)H_−2__ and *k*_Al(L)H_−3__; see Equations (9) and (10)) of the *[Al(L)H_−2_] species. In the calculation of the kinetic parameters characterizing the [Al(CD3A-Bn)]-Cu(II)-citrate reacting system, the third term of the numerator and the second term of the denominator in Equation (12) were neglected due to the lack of the *[Al(L)H_−2_] species.

The *k*_1_ rate constant characterizing the OH^−^ assisted dissociation of [Al(CD3A-Bn)H_−1_]^−^ is about four and fifteen times lower than that of [Al(AMPDA-HB)H_−1_]^−^ and [Al(AMPTA)H_−1_]^−^ intermediates. In the OH^−^ assisted dissociation of the [Al(L)H_−1_]^−^ complexes, the formation of the *[Al(L)H_−2_]^2−^ intermediate presumably takes place by the substitution of the −COO^−^ group with the OH^−^ ion in the inner sphere of Al(III). The spontaneous dissociation of the *[Al(L)H_−2_]^2−^ species presumably occurs by the intramolecular rearrangement of the Al(III) complex and by the stepwise de-coordination of each donor atom and consequent release of the Al^3+^ ion. The fast spontaneous dissociation of *[Al(AMPDA-HB)H_−2_]^2−^ and *[Al(AMPTA)H_−1_]^2−^ intermediates can be ascribed to the less rigid coordination environment for the Al(III) ion provided by two amino-N, one or two carboxylate- and phenolate-O^−^ donor atoms, and two OH^−^ ions, leading to faster intramolecular rearrangements and dissociation processes. However, the spontaneous dissociation (*k*_1_) of the *[Al(AMPDA-HB)H_−2_]^2−^ is significantly slower than that of *[Al(AMPTA)H_−1_]^2−^ due to the stronger interaction of the Al(III) ion with the phenolate-O^−^ donor than that of the carboxylate. Stronger affinity of AMPDA-HB to the Al(III) ion is clearly indicated by the higher *K*_Al(L)H_−2__ value characterizing the formation of *[Al(AMPDA-HB)H_−2_]^2−^ species. As shown in Figure 6, the spontaneous dissociation of the [Al(L)H_−1_] species has a substantial contribution to the dissociation of [Al(CD3A-Bn)H_−1_]^−^ at physiological pH. By taking into account the rate and equilibrium constants presented in Table 2, the half-lives (*t*_1/2_ = ln2/*k*_d_) of the dissociation reactions of [Al(AMPTA)], [Al(AMPDA-HB)], and [Al(CD3A-Bn)] close to physiological conditions (pH 7.4, 25 °C) were calculated and compared with those of [Al(NOTA)]. The dissociation half-life of [Al(CD3A-Bn)] is about two and five times higher than [Al(AMPDA-HB)] and [Al(AMPTA)] due to the slower OH^−^ assisted dissociation of the [Al(L)H_−1_] species at pH 7.4. The *t*_1/2_ value of [Al(AMPDA-HB)] is about three times higher than that of [Al(AMPTA)], highlighting that the substitution of one carboxylate with one phenolate pendant arm in the AMPTA ligand results in the improvement of the kinetic inertness of the Al(III) complex. Although a more appropriate comparison of the kinetic inertness of these complexes with pentadentate ligands with a macrocyclic-like complex would have been Al(NODA)-like systems, the only data available are those of [Al(NOTA)]. Interestingly, the dissociation half-life of [Al(NOTA)] is only about four, eight, and twenty-one times higher than that of [Al(CD3A-Bn)], [Al(AMPDA-HB)], and [Al(AMPTA)]. These findings highlight that the macrocyclic Al(III) complexes with the triazacyclononane derivative ligands have a slightly improved kinetic inertness with respect to those of the Al(III) complexes formed with the open-chain EDTA or CDTA derivatives [20].

### 2.3. Serum Stability of AlF^2+^-Complexes

The Al^19^F complexes were dissolved in an aqueous solution of Seronorm^®^ and ^19^F NMR spectra were recorded at different times in order to determine the stability of the [Al(F)(L)]^−^ ternary complexes. The spectra were recorded every 15 min for the first 3 h and then after 24 h (Figures S8 and S9). No substantial variation in the ^19^F NMR signals’ chemical shift and intensity was observed in 24 h, thus highlighting the excellent stability of the aluminum fluoride ternary complexes in physiological conditions.

## 3. Materials and Methods

### 3.1. General

All chemicals were purchased from Sigma-Aldrich or Alfa Aesar unless otherwise stated and were used without further purification. The ^1^H and ^13^C NMR spectra were recorded using a Bruker Advance III 500 MHz (11.4 T) spectrometer equipped with 5 mm PABBO probes and BVT-3000 temperature control unit. Chemical shifts δ are reported relative to TMS and were referenced using the residual proton solvent resonances. HPLC analyses and mass spectra were performed on a Waters HPLC-MS system equipped with a Waters 1525 binary pump. Analytical measurements were carried out on a Waters XTerra MS C18 (5 μm 4.6 × 100 mm) and on a Waters C18 XTerra Prep (5 μm 19 × 50 mm) for preparative purposes. Electrospray ionization mass spectra (ESI MS) were recorded using an SQD 3100 Mass Detector (Waters), operating in positive or negative ion mode, with 1% *v*/*v* formic acid in methanol as the carrier solvent.

### 3.2. Equilibrium Measurements

The chemicals used for the experiments were of the highest analytical grade. The concentration of the ZnCl_2_ and CuCl_2_ solutions was determined by complexometric titration with standardized Na_2_H_2_EDTA and xylenol orange (ZnCl_2_) and murexid (CuCl_2_) as indicators. Al(NO_3_)_3_ was prepared by dissolving metallic aluminum (99.9%, Fluka) in 6 M HNO_3_ and evaporating off the excess acid. The Al(NO_3_)_3_ residue was dissolved in 0.1 M HNO_3_ solution. The concentration of the Al(NO_3_)_3_ solution was determined by using the standardized Na_2_H_2_EDTA in excess. The excess of the Na_2_H_2_EDTA was measured with standardized ZnCl_2_ solution and xylenol orange as indicator. The H^+^ concentration of the Al(NO_3_)_3_ solution was determined by pH potentiometric titration in the presence of Na_2_H_2_EDTA excess. The concentration of AMPTA, AMPDA-HB, and CD3A-Bn was determined by pH-potentiometric titration in the presence and absence of a large (40-fold) excess of CaCl_2_. The pH-potentiometric titrations were made with standardized 0.2 M NaOH.

The protonation constants of the AMPTA, AMPDA-HB, and CD3A-Bn and those of the Al(III) complexes were determined by pH-potentiometric titration. The metal-to-ligand concentration ratio was 1:1 (the concentration of the ligand was generally 0.002 M). For the pH measurements and titrations, a Metrohm 888 Titrando automatic titration workstation with a Metrohm-6.0234.110 combined electrode was used. Equilibrium measurements were carried out at a constant ionic strength (0.15 M NaNO_3_) in 6 mL samples at 25 °C under magnetic stirring and N_2_ bubbling. The titrations were made in the pH range 1.7–12.0. KH-phthalate (pH = 4.005) and borax (pH = 9.177) buffers were used to calibrate the pH meter. For the calculation of [H^+^] from the measured pH values, the method proposed by Irving et al. was used [34]. A 0.01 M HNO_3_ solution was titrated with the standardized NaOH solution in the presence of 0.15 M NaNO_3_ ionic strength. The differences (*A*) between the measured (pH_read_) and calculated pH (−log[H^+^]) values were used to obtain the equilibrium H^+^ concentration from the pH values measured in the titration experiments (*A* = −0.035). The waiting time between two pH measurements was 60 s. For the equilibrium calculations, the stoichiometric water ionic product (p*K*_w_) was also needed to calculate [OH^−^] values under basic conditions. The V_NaOH_-pH_read_ data pairs of the HNO_3_NaOH titration obtained in the pH range 10.5–12.0 were used to calculate the p*K*_w_ value (p*K*_w_ = 13.78). The protonation and stability constants were calculated with the PSEQUAD program [35].

### 3.3. NMR Experiments

^1^H, ^19^F, and ^27^Al NMR measurements were performed using either a Bruker Avance III (9.4 T) spectrometer, equipped with Bruker Variable Temperature Unit (BVT) and Bruker Cooling Unit (BCU), or a BB inverse *z* gradient probe (5 mm). The formation and protonation processes of [Al(AMPTA)], [Al(AMPDA-HB)], and [Al(CD3A-Bn)] were followed by ^1^H, ^19^F, and ^27^Al NMR spectroscopy at 298 K. For these experiments, 5.0 mM and 4.0 mM aqueous solutions of each Al complex in the presence of 0.15 M NaNO_3_ were prepared (a capillary with D_2_O was used for lock). The pH values of the samples were adjusted with the addition of concentrated NaOH or HNO_3_ solution in the pH range 7–12. The pH range where the complexation equilibria existed and the time needed to reach the equilibria were determined by ^27^Al NMR spectroscopy for the formation of [Al(OH)_4_]^−^. Since the metal exchange of [Al(AMPTA)], [Al(AMPDA-HB)], and [Al(CD3A-Bn)] with [Al(OH)_4_]^−^ is a slow process on the NMR timescale, the stability constants of the complexes were calculated by using the integrals of the ^27^Al NMR signal of the [Al(OH)_4_]^−^ complex. For the calculations of the stability constant, the protonation constant of [Al(AMPTA)], [Al(AMPDA-HB)], and [Al(CD3A-Bn)] obtained by pH-potentiometry and the molar integral values of the ^27^Al NMR signal of the [Al(OH)_4_]^−^ complex were used. The molar integral values of the ^27^Al NMR signal of the [Al(OH)_4_]^−^ complexes were determined by recording the ^27^Al NMR spectra of 1.0, 3.0, 5.0, and 7.0 mM solutions of the [Al(OH)_4_]^−^ complex (pH 12.0, 0.15 M NaNO_3_, 25 °C). The chemical shifts are reported in ppm, relative to DSS for ^1^H and [Al(H_2_O)_6_]^3+^ for ^27^Al as the external standard.

### 3.4. Kinetic Studies

The rates of the exchange reactions taking place between [Al(AMPTA)], [Al(AMPDA-HB)], and [Al(CD3A-Bn)] and Cu(II) in the presence of citrate were studied by spectrophotometry, following the formation of the resulting Cu(II) complexes at 295 nm, with the use of 1.0 cm cells and a PerkinElmer Lambda 365 UV-Vis spectrophotometer at 25 °C in 0.15 M NaCl solution. The concentration of the Al(III) complexes was 0.1 mM, while that of Cu(II) was 10 times higher, to ensure pseudo-first-order conditions. In order to prevent the hydrolysis of Al(III) and Cu(II) ions, the transmetallation reactions were studied in the presence of citrate excess ([Cit]_t_ = 10 and 20 mM). The exchange rates were studied in the pH range 7.0–10.5. For keeping the pH values constant, HEPES and piperazine buffer (0.01 M) were used. The pseudo-first-order rate constants (*k*_d_) were calculated by fitting the absorbance data to Equation (13).

(13) $$A_{t}=\left(A_{0}-A_{p}\right)e^{-k_{d}t}+A_{p}$$

where *A_t_*, *A*_0_, and *A_p_* are the absorbance values at time *t*, the start of the reaction, and at equilibrium, respectively. The calculation of the kinetic parameters was performed by the fitting of the absorbance—time and relaxation rate—time data pairs with the Micromath Scientist computer program (version 2.0, Salt Lake City, UT, USA).

## 4. Conclusions

The semi-rigid pentadentate chelators investigated in this work are among the most promising systems for room temperature [Al^18^F]^2+^ labeling of biomolecules for PET imaging, and thus, the thermodynamic stability and kinetic inertness of their Al(III) complexes were studied in detail. In particular, [Al(AMPDA-HB)] showed the highest thermodynamic stability constant with a log*K*_AlL_ of 18.6, about four orders of magnitude higher than that of [Al(AMPTA)] and [Al(CD3A-Bn)]. With regards to the kinetic inertness, the dissociation half-life of [Al(CD3A-Bn)] is about two and five times higher than [Al(AMPDA-HB)] and [Al(AMPTA)] due to the slower OH^−^-assisted dissociation of the hydroxo-complex ([Al(L)H_−1_]^−1^) at pH 7.4. Moreover, the Al^19^F complexes are shown to be stable with no change in the ^19^F NMR peak in human serum for at least 24 h. All these data confirm that the [Al^18^F]^2+^-labelled pentadentate ligands discussed in this work are well suitable for in vivo PET imaging once conjugated to a biomolecule, with [Al(AMPDA-HB)] showing a much better thermodynamic stability and [Al(CD3A-Bn)] a two-fold higher kinetic inertness. Thus, the conjugation of these pentadentate chelators to selected biomolecules will bring important innovation in the field of AlF-18 radiolabelling, providing a new tool for oncological or immunoPET imaging. Clearly, the in vivo application is essential to determine the real applicability of these chelators with the AlF-18 approach, but the results reported in this work allow for defining the chemical safety of these systems.

## Figures and Tables

**Table 2:** Rate (*k*) and equilibrium (*K*) constants and half-life values (t_1/2_ = ln2/*k*_d_) characterizing the dissociation reactions of the Al(III)-complexes with AMPTA, AMPDA-HB, CD3A-Bn, and NOTA (25 °C).

	[Al(AMPTA)]	[Al(AMPDA-HB)]	[Al(CD3A-Bn)]	[Al(NOTA)] ^a^
I	0.15 M NaCl			1.0 M NaCl
*k*_0_/s^−1^	-	-	(4 ± 1) × 10^−6^	2 × 10^−6^
*k*_1_/M^−1^ s^−1^	164 ± 20	43 ± 5	11 ± 1	6.8 × 10^−6 b^
*k*_2_/M^−2^ s^−1^	(3.4 ± 0.6) × 10^5^	(4 ± 1) × 10^5^	-	-
*K*_Al(L)H_−2__/M^−1^	(1.0 ± 0.2) × 10^8^	(3.2 ± 0.4) × 10^8^	-	1.6 × 10^12 c^
log*K*_Al(L)H_−2__	8.0 (1)	8.5 (1)	-	12.2
*k*_d_/s^−1^ (pH = 7.4)	4.3 × 10^−5^	1.6 × 10^−5^	8.0 × 10^−6^	2 × 10^−6^
*t*_1/2_/h (pH = 7.4)	4.5	12.4	24.1	94

^a^ Ref. [20]; ^b^ *k*_OH_ and ^c^ *K*_Al(NOTA)H_−1__ for [Al(NOTA)] complex.

## Data Availability

The data are available on request from the corresponding authors.

## Supplementary Materials

The following supporting information can be downloaded at: https://www.mdpi.com/article/10.3390/molecules28093764/s1, Figure S1: ^1^H NMR spectra of the Al^3+^-AMPTA system ([Al^3+^] = [AMPTA] = 5.0 mM, 9.4 T, 0.15 M NaNO_3_, 25 °C); Figure S2: ^27^Al NMR spectra of the Al^3+^-AMPTA system ([Al^3+^] = [AMPTA] = 5.0 mM, 9.4 T, 0.15 M NaNO_3_, 25 °C) Figure S3: ^1^H NMR spectra of the Al^3+^-AMPDA-HB system ([Al^3+^] = [AMPDA-HB] = 5.0 mM, 9.4 T, 0.15 M NaNO_3_, 25 °C); Figure S4: ^27^Al NMR spectra of the Al^3+^-AMPDA-HB system ([Al^3+^] = [AMPDA-HB] = 5.0 mM, 9.4 T, 0.15 M NaNO_3_, 25 °C); Figure S5: ^1^H NMR spectra of the Al^3+^-CD3A-Bn system ([Al^3+^] = [CD3A-Bn] = 4.0 mM, 9.4 T, 0.15 M NaNO_3_, 25 °C); Figure S6: ^27^Al NMR spectra of the Al^3+^-CD3A-Bn system ([Al^3+^] = [CD3A-Bn] = 4.0 mM, 9.4 T, 0.15 M NaNO_3_, 25 °C); Figure S7: ^27^Al NMR spectra of the Al^3+^ complexes of ligands AMPTA (*bottom*), AMPDA-HB (*middle*) and CD3A-Bn (*top*) at pH 7 and 25 °C; Figure S8: ^19^F NMR spectra of the [Al(F)(AMPTA)]^−^ complex at different times after addition of human serum ([complex] = 6.6 mM, D_2_O = 0.5 mL, Seronorm = 43 mg, pH 7, 25 °C); resonances from TFA (−76.8 ppm) and AlF_3_ (−123.2 ppm) are also observable; Figure S9: ^19^F NMR spectra of the [Al(F)(AMPDA-HB)]^−^ complex at different times after addition of human serum ([complex] = 4.6 mM, D_2_O = 0.5 mL, Seronorm = 43 °mg, pH 7, 25 °C); resonances from AlF_3_ (−123.2 ppm) are also observable.

## Sample Availability

Samples of the compounds are not available on request from the authors.
