# Supplementary material for: Thermodynamic and Kinetic Stabilities of Al(III) Complexes with N2O3 Pentadentate Ligands
Source: Molecules. 2023 Apr 27;28(9):3764. doi: 10.3390/molecules28093764 (PMC10180113; doi:10.3390/molecules28093764)
Supplement: Supplementary file 1 [file molecules-28-03764-s001.zip › molecules-2324849-supplementary.pdf]

1. Equilibrium studies of [Al(AMPTA)], [Al(AMPDA-HB)] and [Al(CD3A-Bn)]

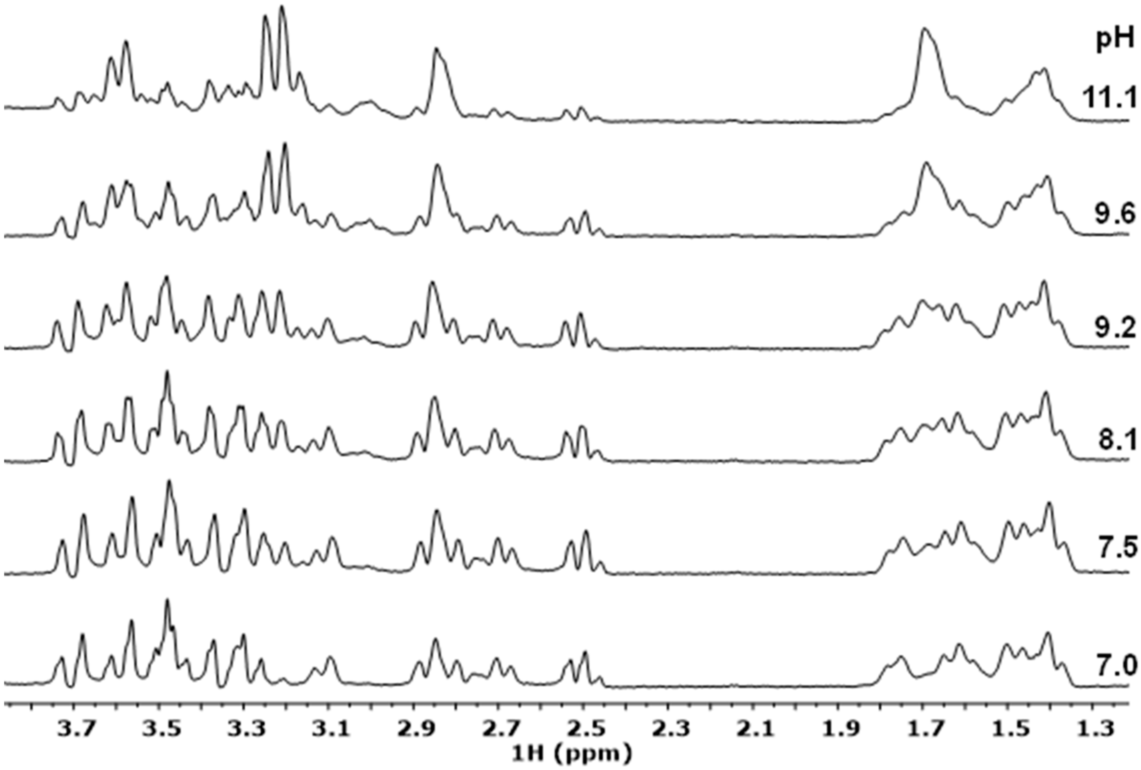

Figure S1. ^{1}H NMR spectra of the Al^{3+} - AMPTA system ($[Al^{3+}]$=[AMPTA]=5.0 mM, 9.4 T, 0.15 M $NaNO_3$, 25 °C).

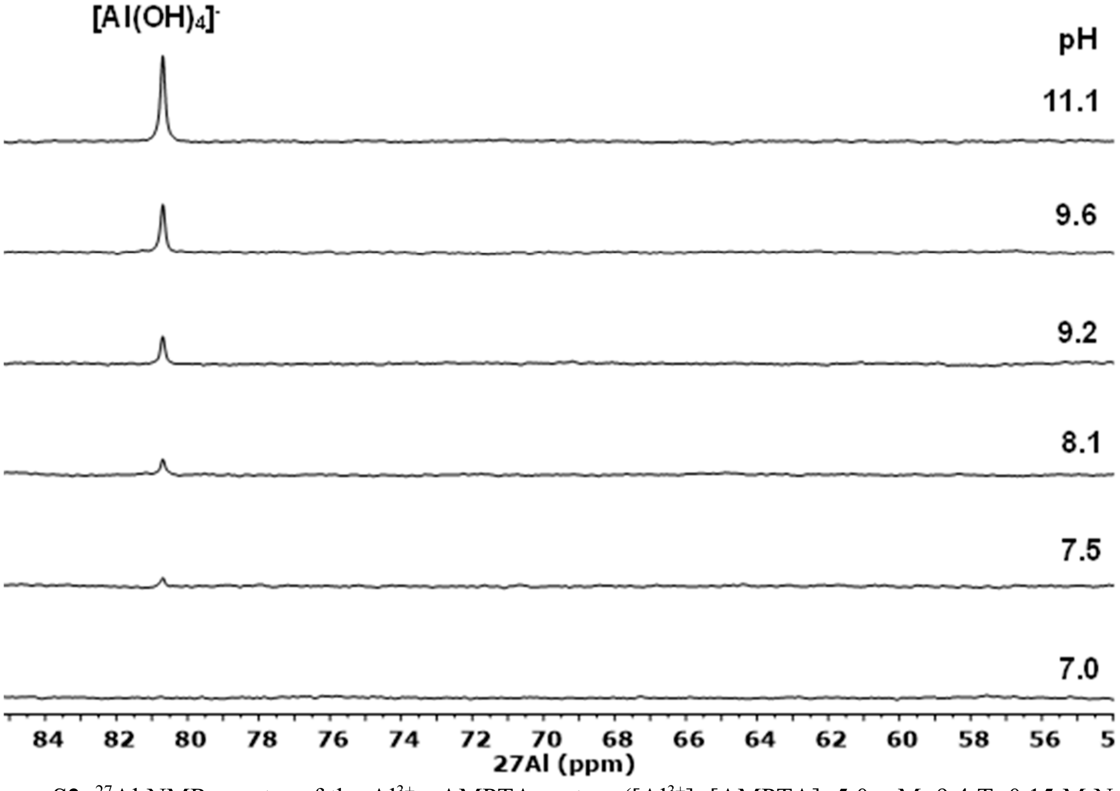

Figure S2. ^{27}Al NMR spectra of the Al^{3+} - AMPTA system ($[Al^{3+}]$=[AMPTA]=5.0 mM, 9.4 T, 0.15 M $NaNO_3$, 25 °C).

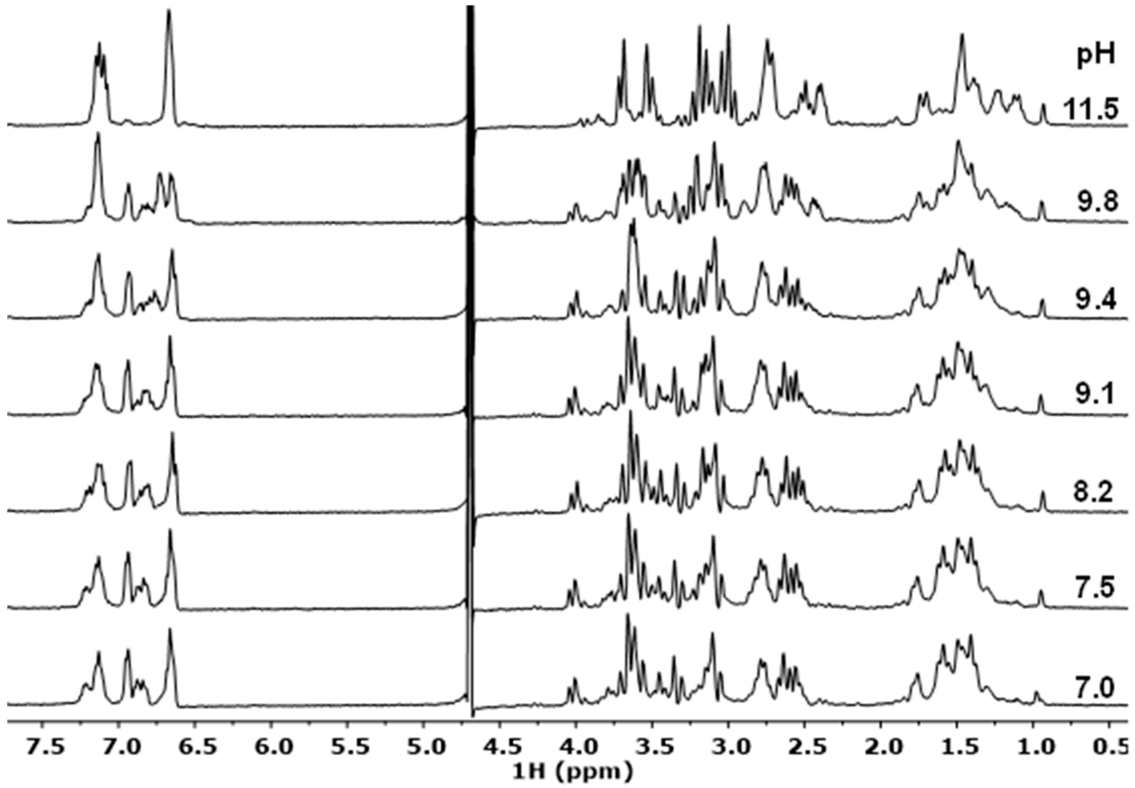

Figure S3. ^{1}H NMR spectra of the Al^{3+} - AMPDA-HB system ($[Al^{3+}]$=[AMPDA-HB]=5.0 mM, 9.4 T, 0.15 M $NaNO_3$, 25 °C).

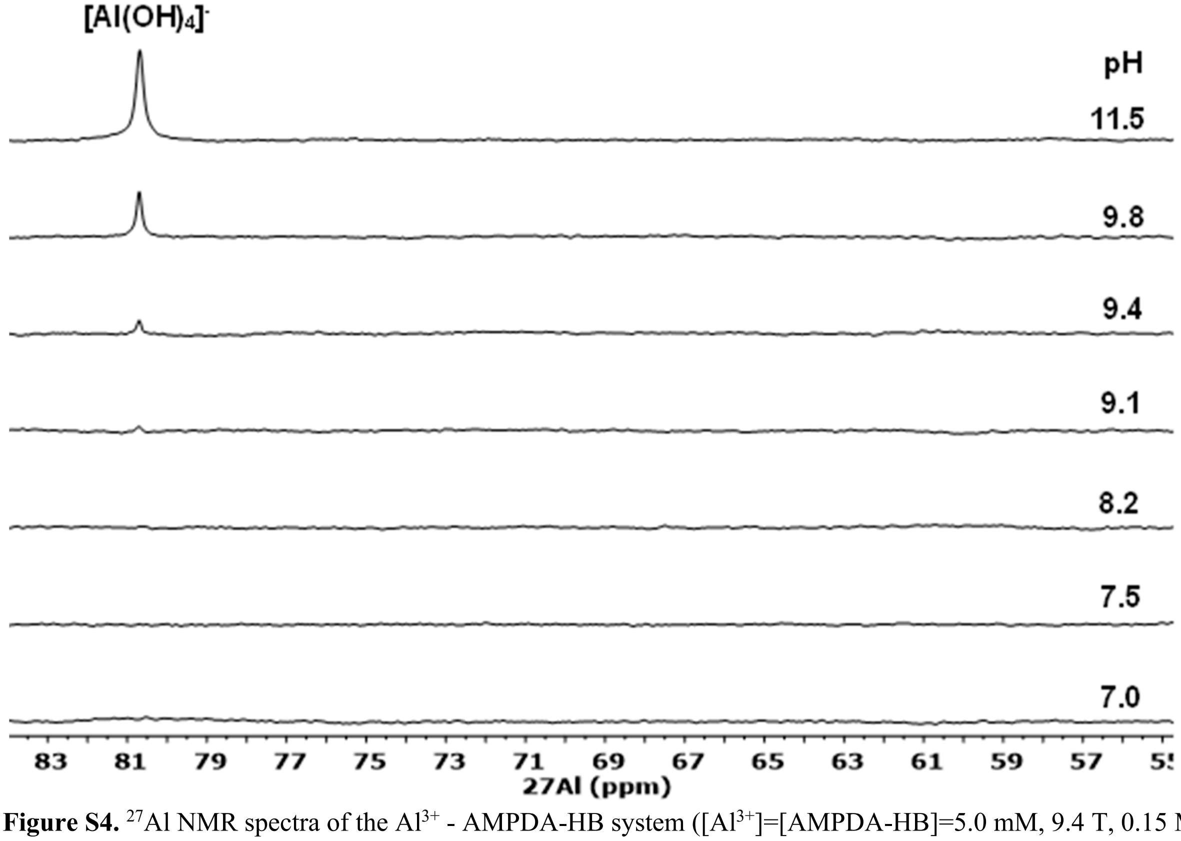

Figure S4. ^{27}Al NMR spectra of the Al^{3+} - AMPDA-HB system ($[Al^{3+}]$=[AMPDA-HB]=5.0 mM, 9.4 T, 0.15 M $NaNO_3$, 25 °C).

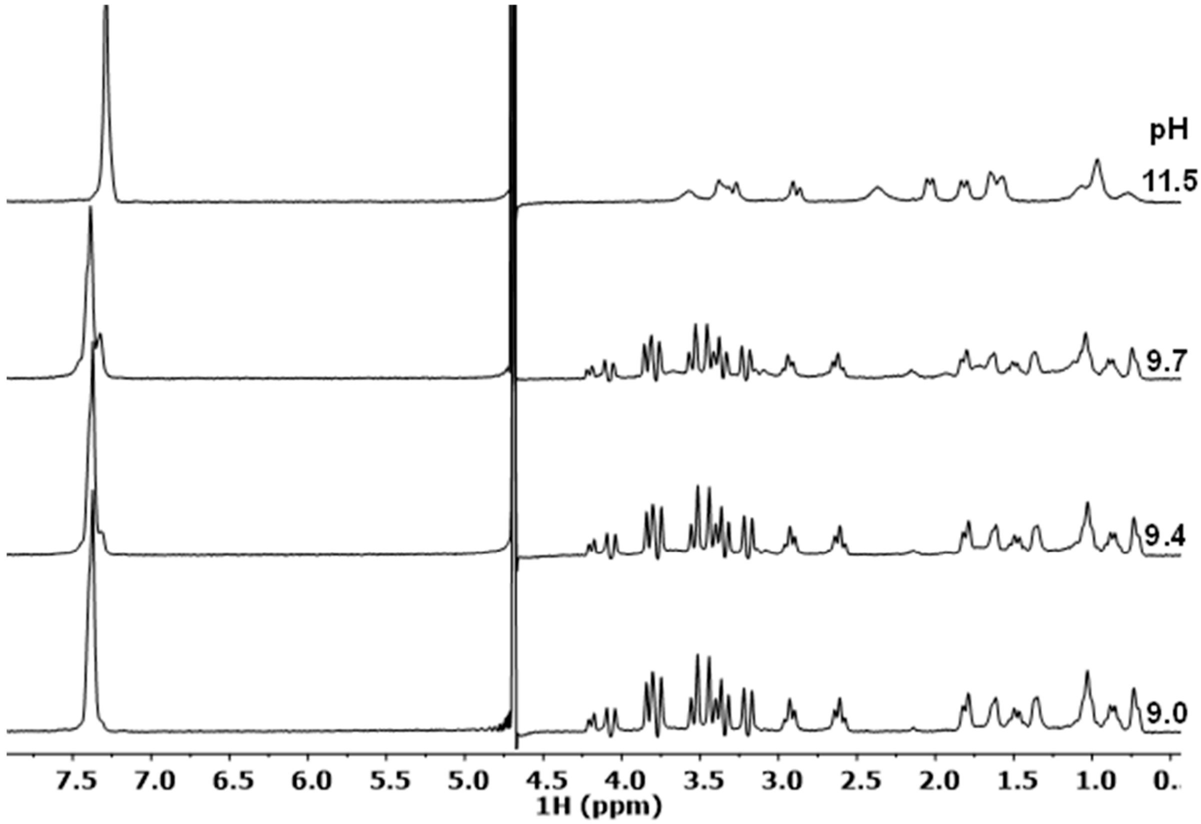

Figure S5. ^{1}H NMR spectra of the Al^{3+} - CD3A-Bn system ($[Al^{3+}]$=[CD3A-Bn]=4.0 mM, 9.4 T, 0.15 M $NaNO_3$, 25 °C).

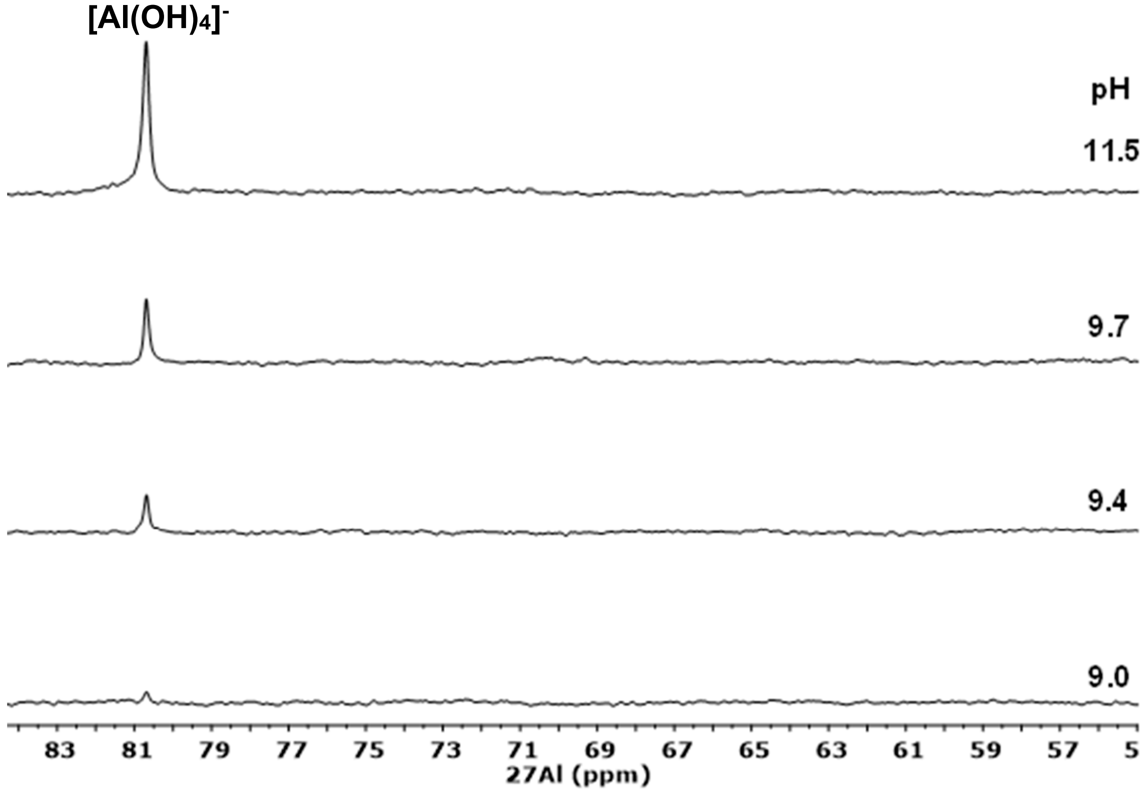

Figure S6. ^{27}Al NMR spectra of the Al^{3+} - CD3A-Bn system ($[Al^{3+}]$=[CD3A-Bn]=4.0 mM, 9.4 T, 0.15 M $NaNO_3$, 25 °C).

2. ^{27}Al NMR spectra

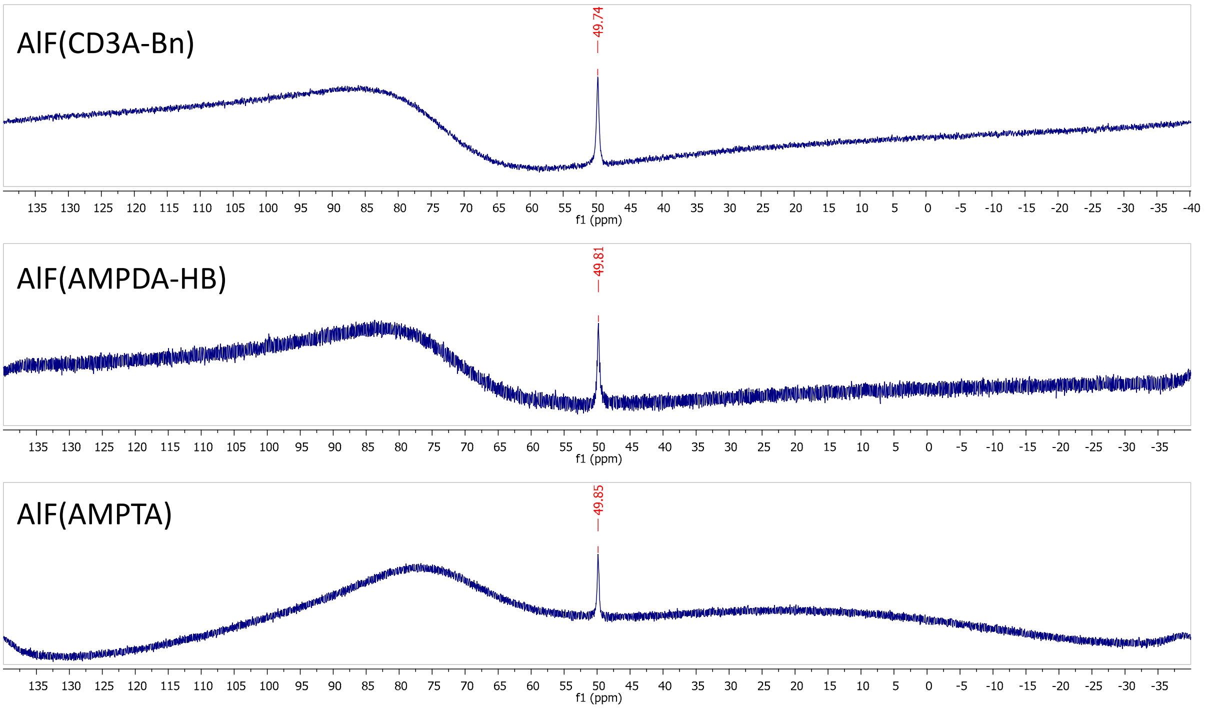

Figure S7. ^{27}Al NMR spectra of the Al^{3+} complexes of ligands AMPTA (*bottom*), AMPDA-HB (*middle*) and CD3A-Bn (*top*) at pH 7 and 25 °C.

3. Stability studies in serum

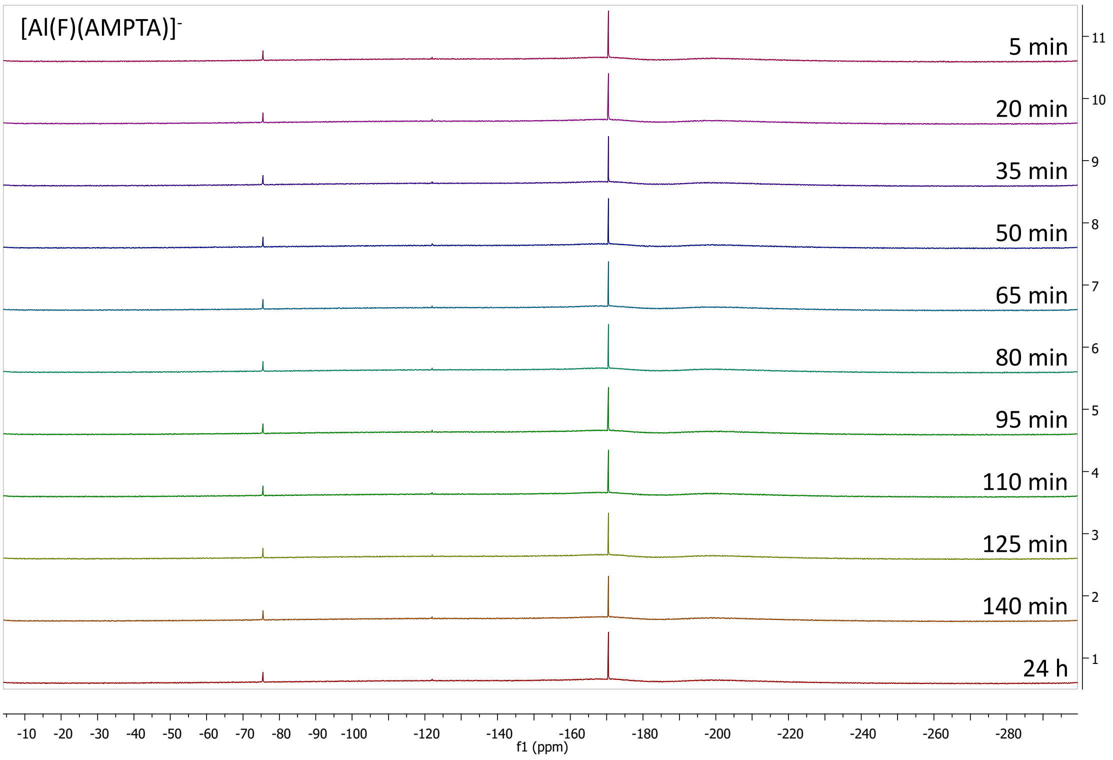

Figure S8. ^{19}F NMR spectra of the $[Al(F)(AMPTA)]^-$ complex at different times after addition of human serum ([complex] = 6.6 mM, D_2O = 0.5 mL, Seronorm = 43 mg, pH 7, 25 °C); resonances from TFA (-76.8 ppm) and AlF_3 (-123.2 ppm) are also observable.

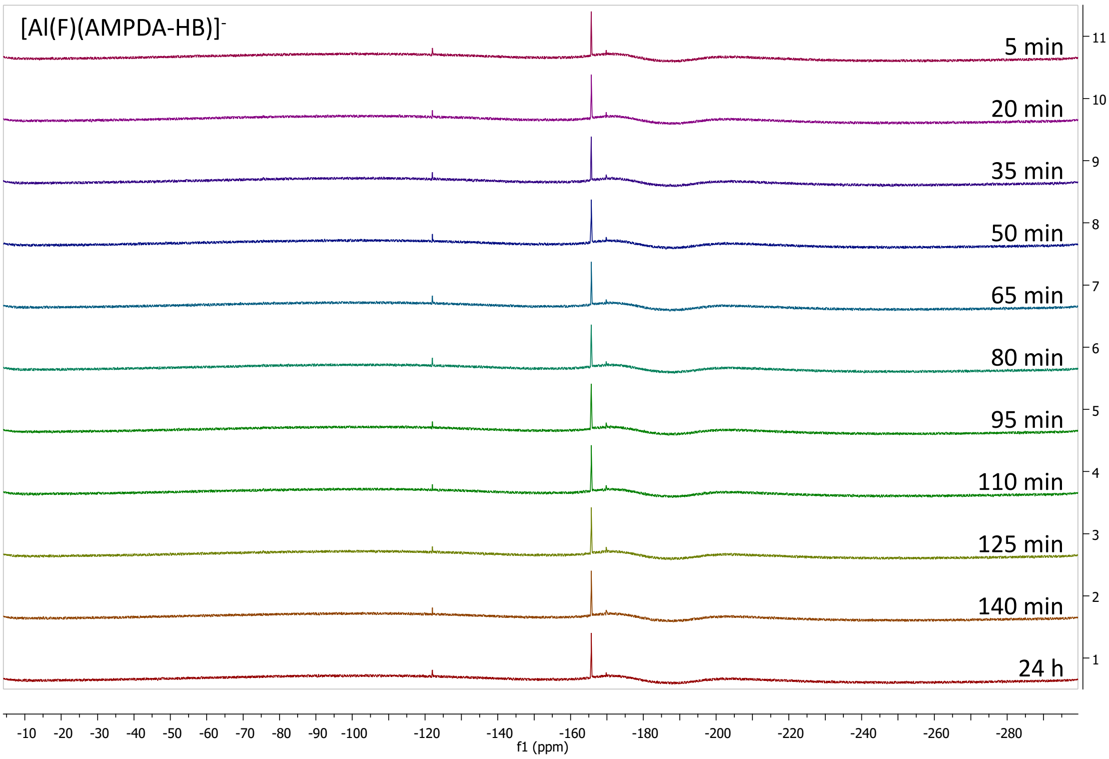

Figure S9. ^{19}F NMR spectra of the $[Al(F)(AMPDA\text{-}HB)]^{-}$ complex at different times after addition of human serum ([complex] = 4.6 mM, D_2O = 0.5 mL, Seronorm = 43 mg, pH 7, 25 °C); resonances from AlF_3 (-123.2 ppm) are also observable.
